# Exploring sex differences in global cancer incidence and mortality and the role of national development level

**DOI:** 10.1016/j.esmorw.2026.100790

**Published:** 2026-10-20

**Authors:** L.A. Hoveling, B.J. Bergink-Voorthuis, J.R.G. van Vlaardingen, R.H.A. Verhoeven, A.D. Wagner, I. Soerjomataram, S. Siesling

**Affiliations:** 1 Department of Research and Development, Netherlands Comprehensive Cancer Organisation (IKNL), Utrecht, The Netherlands; 2 Department of Health Technology and Services Research, Technical Medical Centre, University of Twente, Enschede, The Netherlands; 3 Department of Medical Oncology, Amsterdam UMC location University of Amsterdam, Amsterdam, The Netherlands; 4 Cancer Treatment and Quality of Life, Cancer Center Amsterdam, Amsterdam, The Netherlands; 5 Department of Oncology, Division of Medical Oncology, Lausanne University Hospital (CHUV) and University of Lausanne (UNIL), Lausanne, Switzerland; 6 Cancer Surveillance Branch, International Agency for Research on Cancer, Lyon, France

**Keywords:** cancer incidence, cancer mortality, Human Development Index, sex differences

## Abstract

**Background:**

We analysed sex differences in global cancer incidence and mortality using a national development index to better understand how broader socioeconomic factors may contribute to or mitigate sex differences in cancer outcomes.

**Materials and methods:**

Cancer incidence and mortality data (2012-2017) were obtained from the International Agency for Research on Cancer, covering 43 countries for incidence and 60 for mortality. Only nonsex-specific cancers were included and categorised into 12 groups. Age-standardised incidence rates (ASIR) and age-standardised mortality rates (ASMRs) were calculated, with sex differences analysed using men-to-women ratios (MWRs). Linear regression was used to assess the association between Human Development Index (HDI) and MWRs across cancer types.

**Results:**

Among the cancer types included in this study, cancers of the colorectum, stomach, and lungs showed the highest incidence and mortality. Men are 12-13 times more likely to be diagnosed with oesophageal cancer (MWR ≈ 13), over nine times more likely to die from head and neck cancers, and >6 times more likely to die from lung cancer. Thyroid cancer incidence is up to five times higher in women than in men. No clear link was found between HDI and MWRs in incidence, but HDI relates to mortality and MWRs for several cancer types, with brain and thyroid cancers showing higher male mortality, while lung cancer showing relatively higher female mortality in high-HDI countries.

**Conclusions:**

Sex differences in cancer burden vary substantially across cancer types. National development was associated with sex differences in mortality, but not incidence, suggesting that factors related to health care and survival differ more between countries with different HDIs and contribute to these differences more than exposure to risk factors, which relates more to incidence.

## Highlights

• Real-world registry data reveal sex differences in cancer incidence and mortality.
• Men have higher incidence and mortality for most cancers, except thyroid cancer.
• HDI is associated with sex differences in mortality for specific cancers.
• Health system development and access shape sex gaps in cancer outcomes.

## Introduction

Cancer represents an important global health burden, affecting millions of individuals each year.^1^ Cancer incidence and mortality rates vary widely across different regions and populations,^1^^,^^2^ with varying impact by sex.^1^ Although evidence suggests that men generally experience higher incidence and mortality rates than women,^1^ the magnitude and underlying reasons for these sex differences remain poorly understood.

Sex refers to biological characteristics, whereas sex-related differences in cancer outcomes are also shaped by gender-related factors such as societal roles, health behaviours, and health care access.^3^^,^^4^ While differences in cancer incidence and mortality exist between men and women,^1^ they are often overlooked in public health and clinical practice. Male and female differences (often referred to as sex disparities) can partly be attributed to biological factors such as variations in immune system function, hormonal profiles, molecular characteristics, and body composition.^5^, ^6^, ^7^, ^8^ The role of sex goes, however, beyond biological factors, being also shaped by cultural norms and societal roles, influencing health outcomes in other ways.^9^ Understanding and mapping sex differences in disease incidence and mortality are crucial, as sex affects participation in early detection programs, symptom recognition, health care-seeking behaviour, and treatment decisions^10^ and can point to potential targets for interventions aimed at reducing these disparities.

Furthermore, socioeconomic factors can play a key role in influencing sex differences in cancer. One important macromeasure of country’s socioeconomic development is the Human Development Index (HDI). The HDI reflects a country’s average achievements in health, education, and income^11^ and have been linked to incidence and survival of several cancer types.^12^ Yet, the association between HDI and sex differences in cancer incidence and mortality has not been comprehensively examined using observed registry data.

The aim of our study is threefold: firstly, we will investigate cancer incidence and mortality rates by sex worldwide using population-level data. Secondly, we will investigate global sex differences in these rates. Finally, we will examine the relationship between HDI and sex differences in cancer incidence and mortality rates. Through this comprehensive analysis, our goal was to identify insights to support targeted interventions to reduce sex differences in cancer burden globally.

## Materials and methods

### Data sources

#### Cancer incidence and mortality

Data on cancer incidence and mortality for the period 2012-2017 were obtained from the Cancer over Time database by the International Agency for Research on Cancer (IARC). Incidence and mortality data were included from 43 countries, and mortality data were included from 60 countries. All countries with high-quality incidence and/or mortality data available in the IARC Cancer over Time database were included. These data have undergone quality assessment by IARC to ensure comparability across countries. The Cancer over Time database contains the most recent internationally comparable observed incidence and mortality data available from population-based cancer registries and mortality registries. Because registry data require extensive quality control and validation before publication, particularly in low- and middle-income countries, a time lag between diagnosis and data availability is unavoidable. We therefore considered the Cancer over Time database the most appropriate data source for addressing our study objective of comparing observed sex differences in cancer incidence and mortality across countries. To simplify, we included 12 nonsex-specific cancer types or groups, namely head and neck (C00-14 and C32), oesophageal (C15), stomach (C16), colorectum (C18-C21), pancreas (C25), liver and bile ducts (C22-C24), lung (C33-C34), skin melanoma (C43), kidney (C64), bladder (C67), brain and central nervous system (CNS; C70-C72), and thyroid (C73) cancers (Supplementary Table S1, available at https://doi.org/10.1016/j.esmorw.2026.100790).^13^ Cancer site groupings followed the predefined classifications used in the IARC Cancer over Time database. Age-standardised incidence rates (ASIR) and age-standardised mortality rates (ASMRs) were calculated for these predefined cancer groups rather than by summing rates across individual cancer sites.

#### National development level

National development level was measured per country with the HDI. Data on HDI were obtained from United Nations statistical reports covering the period from 1992 to 2012, allowing for a lag time between exposure to risk factors and the occurrence of cancer. HDI measures key dimensions of human development, namely life expectancy, education level, and national income.^11^ For the HDI, a value closer to 1 indicates a higher national development level.

### Statistical analysis

#### Cancer incidence and mortality

Incidence rates (IRs) were obtained based on cancer diagnoses between 2012 and 2017 and presented as ASIRs per 100 000.^14^ Mortality rates (MRs) were calculated by dividing the number of cancer deaths in each country by population at risk over the same period. Both IRs and MRs were age standardised according to the Segi-Doll World Standard Population to account for differences in age distribution between countries and between men and women, allowing meaningful comparisons across populations. ASIRs and ASMRs were visualised in a heatmap, with darker areas indicating higher rates.

#### Global sex differences

For the aforementioned ASIRs and ASMRs, men-to-women ratios (MWRs) were calculated to measure sex disparity (Supplementary Appendix A, available at https://doi.org/10.1016/j.esmorw.2026.100790). An MWR >1 indicates higher incidence or mortality rates among men, while an MWR <1 suggests higher rates among women.

#### National development level and sex differences

Linear regressions with MWR as an outcome variable and HDI as an independent variable were calculated for each of the 12 cancer categories and for ASIR and ASMR separately. Normal distribution of residuals was checked visually. If residuals were not normally distributed, MWR values equal to 0 were removed, and log transformation was applied to the outcome variable. To facilitate interpretation on the HDI scale (ranging from 0 to 1), coefficients represent the change in the MWR for a 0.1 increase in the HDI. In those cases where log transformation was applied, exp(B) was reported, which reflects the multiplicative change in MWR for a 1-unit increase in the HDI. All analyses were carried out using the R software, version 4.4.3 (R Foundation for Statistical Computing, Vienna, Austria). *P* values <0.05 were statistically significant.

## Results

### Cancer incidence and mortality

#### Incidence rates

Colorectum cancers, lung cancers, and melanomas exhibited the highest IRs for both men and women (Figure 1). However, these ASIRs vary across countries. For example, lung cancer showed highest rates in Turkey, Belarus, and Croatia. In these countries, ASIRs are higher than 50 per 100 000 men. Countries like India, Kuwait, and Qatar report substantially lower ASIRs, with <10 per 100 000 individuals for both sexes. CNS and oesophageal cancers showed lower ASIRs in general. For example, CNS cancer rates had a maximum of 20 per 100 000 women and 16 per 100 000 men, with even lower rates observed for oesophageal cancer.

**Figure 1:**
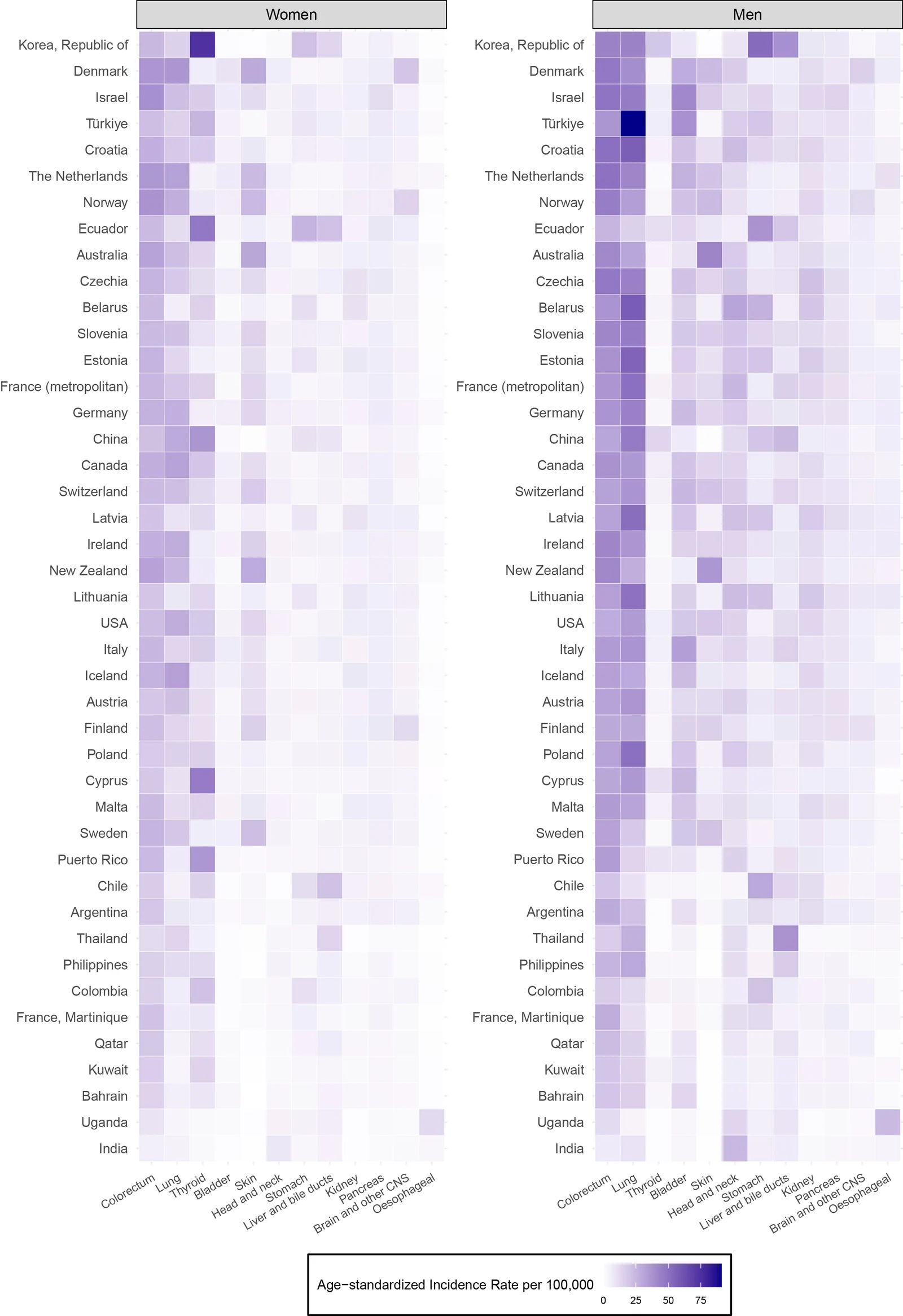
**Heatmap of cancer incidence rates by country, sex, and cancer category with age-standardised incidence rates per 100 000 individuals.** Note: ranges of legend are based on maximum and minimum age-standardised incidence rate in dataset. CNS, central nervous system.

#### Mortality rates

Colorectum, stomach, and lung cancers showed the highest ASMRs across all countries for both men and women (Figure 2). Countries with the highest mortality rates for these cancers are Poland, Hungary, Serbia, Armenia, and Croatia. For instance, in Poland and Hungary, lung cancer mortality rates are 50 and 65 per 100 000 men, respectively, and 18 and 29 per 100 000 women, respectively. Guatemala, Guyana, and Ecuador showed lower rates ranging from 4 to 6 per 100 000 for men and 3 to 4 per 100 000 for women.

**Figure 2:**
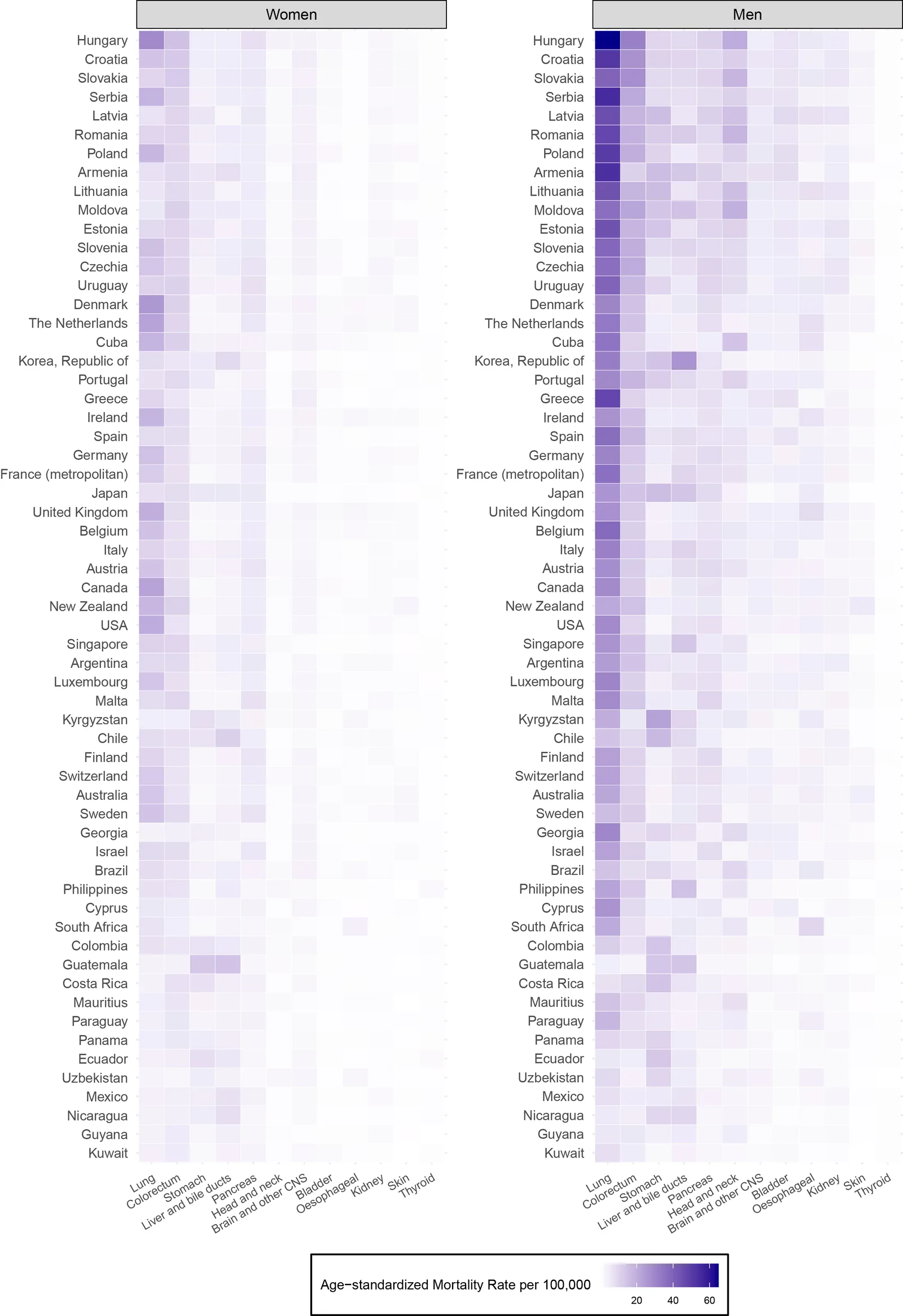
**Heatmap of cancer mortality rates by country, sex, and cancer category by age-standardised mortality rates given per 100 000 individuals.** Note: ranges of legend are based on maximum and minimum age-standardised mortality rate in dataset. CNS, central nervous system.

### Sex differences in incidence and mortality

#### Incidence MWRs

Overall, cancers of the bladder, colorectum, oesophagus, head and neck, liver, lung, and stomach and skin melanoma are more common in men. In contrast, thyroid cancer is more incident in women. Differences in cancer incidence between men and women were most notable for oesophageal, head and neck, lung, and thyroid cancers. These cancers were selected because they showed the largest variation in MWRs across countries, reflecting substantial sex-related differences that may be linked to behavioural, environmental, or biological factors. For oesophageal cancer, Qatar was the only country where more women were diagnosed than men (Figure 3A). In most African and Asian countries, MWRs ranged between 1 and 2, while in countries like Thailand and China and parts of Europe, men were over five times more frequently diagnosed than women. In Belarus and Korea, MWRs reached values as high as 12 to 13. For head and neck cancer, eight countries had MWRs exceeding four, with Poland, Croatia, Latvia, Lithuania, and Belarus reporting values over five (Figure 3B). Lung cancer also showed pronounced sex differences; in 10 countries, the incidence in men was more than three times higher than in women, with Belarus showing an MWR above nine (Supplementary Figure S1, available at https://doi.org/10.1016/j.esmorw.2026.100790). In contrast, thyroid cancer was more common among women. In Poland, Argentina, Chile, Qatar, and Colombia, women were diagnosed about five times more often than men (Supplementary Figure S2, available at https://doi.org/10.1016/j.esmorw.2026.100790). For other cancer types including bladder, CNS, colorectum, kidney, liver and bile duct, pancreas, and stomach cancers and skin melanoma, male-to-female incidence ratios were generally closer to 1, indicating smaller sex differences.

**Figure 3:**
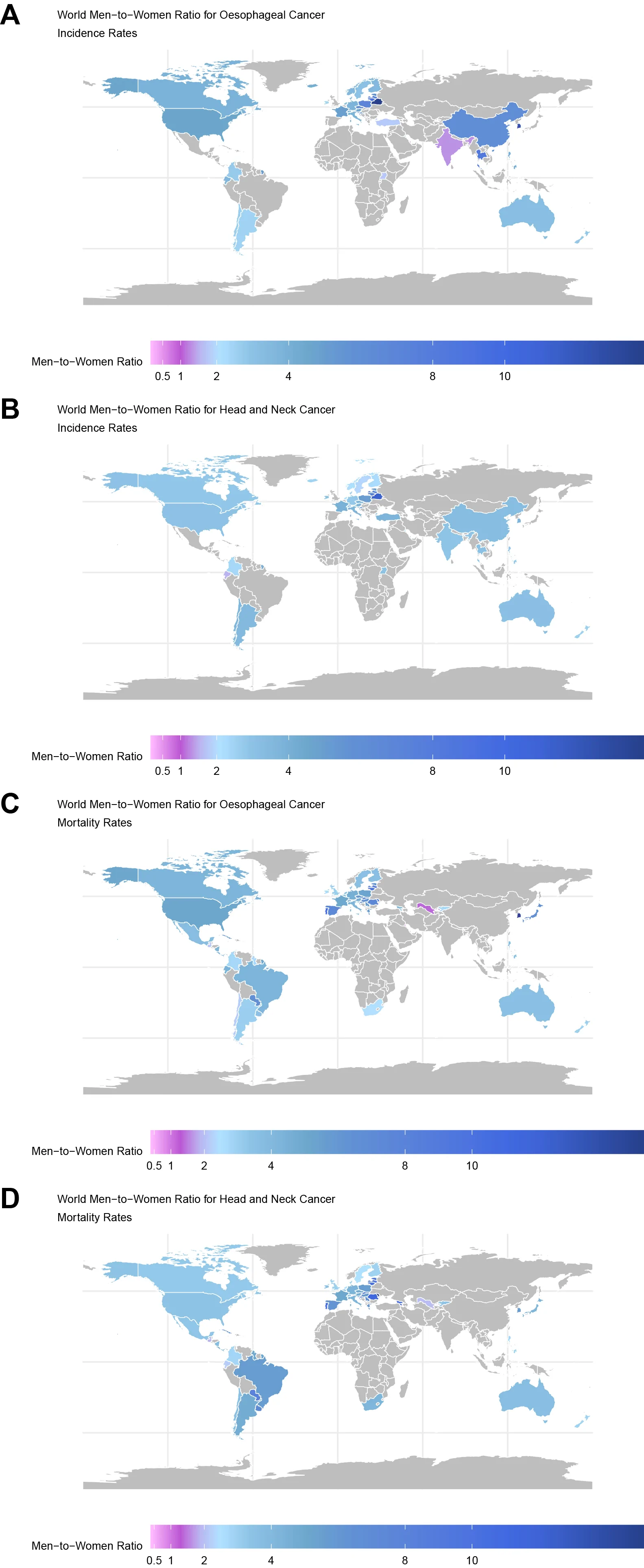
**Global sex disparity for oesophageal and head and neck cancer incidence and mortality (A-D).** MWR <1 is represented by a pink–violet colour spectrum, indicating higher incidence or mortality rates in women. A violet–blue colour spectrum indicates an MWR >1, reflecting higher incidence or mortality rates in men. Subfigures show the following: (A) oesophageal cancer incidence, (B) head and neck cancer incidence, (C) oesophageal cancer mortality, and (D) head and neck cancer mortality. MWR, Men-to-women ratio.

#### Mortality MWRs

Overall, cancers of the colorectum, oesophagus, head and neck, kidney, liver and bile ducts, lung, and stomach showed higher mortality among men. Other cancers showed comparable mortality in men and women. Differences between men and women in cancer mortality are particularly pronounced in oesophageal, head and neck, and lung cancers. In Estonia, Hungary, Paraguay, Japan, Cyprus, Spain, Romania, Croatia, Latvia, Slovakia, and Lithuania, men are more than six times more likely to die from oesophageal cancer than women (Figure 3C). In Moldova, Portugal, and the Republic of Korea, this risk is even over 10 times higher for men. For head and neck cancer, in Croatia, Estonia, Latvia, Armenia, Slovakia, Lithuania, Portugal, Georgia, Romania, and Moldova, men are more than nine times more likely to die from this cancer than women (Figure 3D). Regarding lung cancer, the sex disparity is somewhat smaller, but there are still several countries where men are more than six times more likely to die from lung cancer than women (Supplementary Figure S3, available at https://doi.org/10.1016/j.esmorw.2026.100790). These countries include Moldova, Latvia, Lithuania, Armenia, and Georgia. For thyroid cancer, the differences in mortality between men and women are less pronounced (Supplementary Figure S4, available at https://doi.org/10.1016/j.esmorw.2026.100790). However, in Luxembourg and Guyana, men are more than two times more likely to die from this type of cancer than women. For bladder, CNS, colorectum, thyroid, kidney, liver and bile duct, pancreas, and stomach cancers and skin melanoma, the mortality differences between men and women are minor.

### National development level and sex differences

#### National development level and incidence MWRs

No significant associations between HDI and incidence MWRs were found for any cancer type (Table 1). For brain and CNS, colorectum, thyroid, and pancreatic cancers, the effect of HDI is very weak. For other cancer types, the effect was found to be slightly stronger, although not significant. In general, a higher HDI was associated with a higher MWR, indicating higher incidence for men compared with women, except for head and neck cancer and for lung cancer.

**Table 1:** Linear regression coefficients representing the relationship between the HDI and the age-standardised incidence MWR

Cancer category	Incidence MWR	Incidence log(MWR)
	HDI, B (95% CI)^a^	HDI, exp(B) (95% CI)^b^
Bladder		2.16 (0.97-4.79)
Brain and other CNS	0.01 (−0.06 to 0.08)	
Colorectum	0.00 (−0.06 to 0.06)	
Thyroid	0.01 (−0.01 to 0.03)	
Oesophageal		2.59 (0.55-12.59)
Head and neck		0.72 (0.24-2.18)
Kidney	0.07 (−0.01 to 0.15)	
Liver and bile ducts	0.13 (−0.05 to 0.30)	
Lung		0.42 (0.09-2.07)
Pancreas	0.02 (−0.05 to 0.09)	
Skin melanoma	0.05 (−0.02 to 0.11)	
Stomach	0.08 (−0.02 to 0.18)	

B, β coefficient of the association between HDI and age-standardised incidence men-to-women ratio.

CI, confidence interval; CNS, central nervous system; HDI, Human Development Index; MWR, men-to-women ratio.

a Coefficients should be interpreted as the change in the MWR for a 0.1-unit increase in the HDI.

b Coefficients should be interpreted as the multiplicative change in the MWR for a 1-unit increase in the HDI.

#### National development level and mortality MWRs

Regarding mortality, HDI was positively associated with MWRs for CNS and thyroid cancers (Table 2). A higher HDI was linked to a relatively higher mortality among men than in women. HDI was also negatively associated with MWRs for lung cancer, indicating that a higher HDI is linked with a relatively higher mortality among women than in men. Positive, although not significant, associations were also observed for liver and bile duct, bladder, and oesophageal cancer. Negative associations were observed for head and neck cancers, kidney cancers, and skin melanoma. For the other cancer types, the associations with HDI were not evident.

**Table 2:** Linear regression coefficients representing the relationship between the HDI and the age-standardised mortality MWR

Cancer categories	Mortality MWR	Mortality log(MWR)
	HDI, B (95% CI)^a^	HDI, exp(B) 95% CI)^b^
Bladder		1.30 (0.28-6.07)
Brain and other CNS	0.08 (0.01-0.16)∗	
Colorectum	0.03 (−0.10 to 0.17)	
Thyroid	0.22 (0.12-0.33)∗	
Oesophageal		1.94 (0.25-15.02)
Head and neck		0.44 (0.06-3.33)
Kidney		0.23 (−1.63 to 2.09)
Liver and bile ducts	0.22 (−0.06 to 0.49)	
Lung		0.12 (0.01-0.91)∗
Pancreas	−0.03 (−0.14 to 0.09)	
Skin		0.87 (0.35-2.16)
Stomach	−0.03 (−0.19 to 0.13)	

B, β coefficient of the association between HDI and age-standardised mortality men-to-women ratio.

CI, confidence interval; CNS, central nervous system; HDI, Human Development Index; MWR, men-to-women ratio.

a Coefficients should be interpreted as the change in the MWR for a 0.1-increase in HDI.

b Coefficients should be interpreted as the multiplicative change in the men-to-women ratio for a 1-unit increase in the HDI.

∗ *P* < 0.05.

## Discussion

Our study analysed global cancer incidence and mortality, highlighting sex differences and the role of national development. Men showed higher rates for most cancers, especially oesophageal, head and neck, lung, and bladder cancers, while thyroid cancer is more common in women. Some countries show extreme sex gaps, such as oesophageal cancer in Belarus and the Republic of Korea. We found no statistically significant association between HDI and sex differences in cancer incidence, whereas HDI was associated with sex differences in mortality for several cancer types, including CNS, thyroid, and lung cancers. These findings illustrate that sex differences in cancer burden cannot be attributed to a single mechanism but instead arise from the interaction between biological factors, behavioural exposures, and health care systems.

### Cancer incidence and mortality across countries

Our findings for the global cancer incidence and mortality pattern by cancer categories are consistent with several recent studies. For instance, a comprehensive analysis of the cancer burden in Europe reported the high incidence of colorectum, lung, and skin (melanoma) cancers in Northern European countries, including Denmark and the UK.^1^ This study also emphasises the variability within Europe, where Southern European countries, such as Italy and Spain, show lower ASIRs.^15^ Further, studies focusing on sub-Saharan Africa have reported substantially lower ASIRs for cancers such as colorectum and lung cancers than those on Europe and North America.^16^ Similarly, low rates were also seen in India. This regional difference could be attributed to variations in lifestyle factors, limited screening programs, health care access, or limited health care infrastructure. Moreover, in India, similar trends were observed, with lower IRs, for colorectum and lung cancers. These regional differences in cancer burden underscore the need for context-specific prevention, early detection, and health care strategies tailored to local risk profiles and resource availability.

### Sex differences in incidence and mortality

Sex differences in incidence and mortality are particularly pronounced in oesophageal, head and neck, lung, and thyroid cancers, with men generally showing much higher IRs and MRs except for thyroid cancer, which is more common in women. For some cancers, such as bladder, CNS, colorectum, thyroid, kidney, liver and bile duct, pancreas, skin, and stomach cancers, the incidence and mortality differences between men and women are small. Our findings are consistent with existing literature that underscores the persistent sex differences in cancer outcomes and occurrence.^13^^,^^17^ For instance, Siegel et al.^13^ emphasises that men have higher ASIRs and ASMRs for most cancers, particularly oesophageal, head and neck, lung, and urinary tract cancers,^13^ which are strongly associated with tobacco use. Sex differences in tobacco consumption remain an explanatory factor for higher incidence and mortality in men for tobacco-related cancers such as lung, head and neck, and oesophageal cancers. However, regional variations in these disparities are notable, with some countries exhibiting extreme MWRs (e.g. men were 12-13 times more likely to develop oesophageal cancer in Belarus and the Republic of Korea). Historically, men have higher rates of smoking, contributing to their higher risk for these cancers. However, as noted in recent trends, tobacco use among women has been increasing in many regions, potentially narrowing these disparities over time. This highlights the critical need for targeted tobacco cessation programs, particularly for men, who remain at higher risk. Such programs could also pre-emptively address the rising smoking rates among women to mitigate rising future cancer burdens.^18^, ^19^, ^20^ Further, Siegel et al.^13^ reported that women have higher rates of endocrine-related cancers, such as thyroid cancer, while sex differences were small for cancers such as colorectum and skin cancers.^13^ Similarly, studies across time and age groups show that men consistently have higher IRs for cancers such as CNS, oesophageal, liver, lung, and urinary tract cancers, whereas women are more affected by endocrine cancers.^17^ The low MWRs observed for colorectum and skin cancers may reflect more equal exposure to risk factors and similar access to screening programs, especially in high-HDI countries. In contrast, the higher incidence of thyroid cancer in women may be explained by both biological and diagnostic factors. Oestrogen may play a role in thyroid cell proliferation, and women are more likely to undergo health checks where incidental thyroid nodules are detected, potentially leading to overdiagnosis. These findings suggest that while some sex differences reflect true biological differences, others may result from differences in health care utilisation, with implications of how screening and diagnostic practices are applied across sexes.

### National development level and sex differences

No statistically significant associations were observed between HDI and incidence MWRs for any cancer type. Yet, HDI showed a positive association with mortality MWRs for CNS and thyroid cancers, indicating relatively higher male mortality in higher HDI countries, while lung cancer-related mortality and MWRs were negatively associated with HDI, suggesting relatively higher female mortality in higher HDI settings. An earlier study has noted that sex differences in cancer outcomes can be influenced by individual socioeconomic status (SES), with higher SES often associated with more pronounced sex differences.^21^ The increased MWRs in regions with higher HDI levels for certain cancers may reflect greater sex-based differences in exposure to risk factors, health care access, or health behaviours. For instance, although alcohol consumption is prevalent across all socioeconomic groups, studies suggest that harmful or heavy drinking is more common in lower SES groups, particularly among men.^22^ This may contribute to the elevated MWRs observed in alcohol-related cancers such as liver, oesophageal, and head and neck cancers. For colorectum, liver, and skin cancers, the effects of HDI on incidence and mortality MWRs were very weak or nonsignificant, possibly reflecting more equitable health care access across sexes in these contexts.^23^ These findings emphasise the complex impact of distant determinant such as national development in shaping sex differences in cancer outcomes, influenced by a combination of biological, behavioural, and sociocultural factors. One possible explanation for the absence of an association between HDI and sex differences in incidence, while associations were observed for mortality, is that cancer incidence mainly reflects exposure to biological, behavioural, and environmental risk factors, which may not systematically vary with HDI in relation to sex. In contrast, mortality is also influenced by access to health care, early diagnosis, treatment availability, and survival, all of which are more likely to differ according to a country’s level of development. Consequently, national development may have a greater impact on sex differences in cancer mortality than on incidence.

A strength of our study is the utilisation of a large and diverse dataset, including cancer incidence and mortality data from 43 and 60 countries and socioeconomic data covering multiple years, enhancing the generalisability of the findings across different regions and populations. Importantly, the cancer data are based on observed incidence and mortality data from population-based cancer and mortality registries rather than modelled estimates, allowing direct comparison of observed sex differences across countries. By analysing 12 cancer groups using a uniform methodology across countries, our study allows for a consistent and comparative analysis of sex differences across a broad spectrum of cancers and regions, facilitating the recognition of global and regional patterns. A limitation of our study is that the quality and completeness of the data may vary. Variations in data reporting can impact the accuracy, timeliness, and reliability of the findings. For rare cancers in countries with relatively small populations, sex ratios may be less stable because of limited case numbers. Another limitation is that incidence and mortality data were available for different sets of countries. Consequently, comparisons between the incidence and mortality analyses should be interpreted with caution. Nevertheless, each analysis included all countries for which high-quality data were available, and HDI was analysed separately within each dataset. Information on factors such as health care access, cultural differences, and hormonal, biological, and lifestyle influences related to sex would be valuable for a better understanding of the causes behind the observed differences. We used the Segi and Doll World Standard Population to ASIRs and ASMRs. Given the increasing proportion of older adults in many populations, future analyses may consider more recent standard populations that better reflect contemporary age distributions. Although our study includes data from numerous countries, most countries are high-income countries, which may limit the generalisability of the findings to lower-income countries or regions. Future studies using global estimation datasets such as the Global Canser Observatory (GLOBOCAN) may complement our findings by providing broader geographical coverage and more recent estimates. The more recent GLOBOCAN estimates provide additional context for assessing whether the sex differences observed in our registry-based data remain evident in more recent estimates. However, these comparisons should be interpreted with caution because GLOBOCAN estimates are based on modelling rather than directly observed registry data and are therefore not directly comparable with the data used in our study. Analyses using consistent data sources and methodology across multiple time periods would be needed to formally assess temporal changes in these sex differences. Finally, using HDI as a proxy for national development may obscure important individual-level socioeconomic factors, such as income, education, and occupation, which could more directly influence sex differences in cancer incidence and mortality. Our analyses were intentionally limited to HDI as a summary indicator of national development to address the primary study objective. HDI represents only one dimension of national development. Other indicators, including health care expenditure, Universal Health Coverage, gross domestic product, and health system performance, may also contribute to sex differences in cancer outcomes. Future studies should investigate these factors simultaneously using multivariable approaches and explore potential nonlinear associations.

### Conclusions

Our findings indicate global sex differences in cancer incidence and mortality, with men generally exhibiting higher rates for most cancers, particularly oesophageal, head and neck, and lung cancers, while thyroid cancer remains more common in women. These disparities vary considerably across countries and are especially pronounced for tobacco-related cancers. Although no significant associations were found between HDI and incidence MWRs, our findings indicate that national development influences sex differences in cancer mortality for specific cancer types, such as CNS, thyroid, and lung cancer, suggesting that the impact of HDI on sex differences is not uniform but varies by cancer site. Our findings underscore the multifactorial nature of these disparities, shaped by biological, behavioural, and sociocultural factors. The observed high MWRs for tobacco-related cancers, particularly in men, underline the need for sex-specific prevention strategies such as targeted tobacco control. Similarly, the high incidence of thyroid cancer in women, likely partly driven by overdiagnosis, points to the importance of refining diagnostic guidelines. The variation in mortality disparities by HDI suggests that health system development and access play a role in shaping sex gaps in cancer outcomes. Targeted prevention strategies, equitable access to health care, and sex-sensitive cancer control policies could help to reduce these inequalities globally.

## Supplementary data

Supplementary Figures C1-C4
