# Supplementary material for: Exploring sex differences in global cancer incidence and mortality and the role of national development level
Source: ESMO Real World Data Digit Oncol. 2026 Oct 20;14:100790. doi: 10.1016/j.esmorw.2026.100790 (PMC13717883; doi:10.1016/j.esmorw.2026.100790)
Supplement: Supplementary file 1 [file mmc1.docx]

**APPENDIX A**. Description of the calculation of the incidence and mortality men-to-women rates

$$\begin{aligned} Incidence MWR=\frac{Incidence Rate Men}{Incidence Rate Women} \#\left( A.1 \right) \end{aligned}$$

$$\begin{aligned} Mortality MWR=\frac{Mortality Rate Men}{Mortality Rate Women}\#\left( A.2 \right) \end{aligned}$$

**APPENDIX B. SUPPLEMENTARY TABLES**

**Table B1**. Categorization of cancer types

| **Cancer Category** | **Cancer Type** | **ICD-10 Codes** |
| --- | --- | --- |
| Head and neck | Lip | C00 |
|  | Tongue | C01-02 |
|  | Mouth | C03-06 |
|  | Salivary glands | C07-08 |
|  | Tonsil | C09 |
|  | Other oropharynx | C10 |
|  | Nasopharynx | C11 |
|  | Hypopharynx | C12-13 |
|  | Pharynx unspecified | C14 |
|  | Larynx | C32 |
| Oesophageal | Esophagus | C15 |
| Stomach | Stomach | C16 |
| Colorectum | Colorectum | C18-21 |
| Pancreas | Pancreas | C25 |
| Liver and bile ducts | Liver | C22 |
|  | Gallbladder | C23-24 |
| Lung | Lung (incl. trachea and bronchus) | C33-34 |
| Skin | Melanoma of skin | C43 |
| Kidney | Kidney | C64 |
| Bladder | Bladder | C67 |
| Brain and central nervous system | Brain, nervous system | C70-72 |
| Thyroid | Thyroid | C73 |

**APPENDIX C. SUPPLEMENTARY FIGURES**

**Figure C1**. Global sex disparity for lung cancer incidence

**Figure C2**. Global sex disparity for thyroid cancer incidence

**Figure C3**. Global sex disparity for lung cancer mortality

**Figure C4**. Global sex disparity for thyroid cancer mortality
